# Incorporating a Veteran’s lived and living expertise of sex work and substance use into clinician education

**DOI:** 10.1186/s12909-026-09113-x

**Published:** 2026-04-06

**Authors:** Andie Ruggles, Earl Aguirre, Donald Tietjen, Tessa Rife-Pennington

**Affiliations:** 1 Social Work Service, San Francisco VA Health Care System, 401 3rd St, San Francisco, CA 94107 USA; 2 Lived Experience Educator, San Francisco, CA USA; 3 Pharmacy Service, San Francisco VA Health Care System, 4150 Clement St (119), San Francisco, CA 94121 USA; 4 Department of Clinical Pharmacy, School of Pharmacy, University of California, San Francisco, San Francisco, CA USA; 5 Department of Pharmacy Practice, Thomas J. Long School of Pharmacy, University of the Pacific, San Francisco, CA USA

**Keywords:** Veterans, Sexual Health, Sex Work, Substance-Related Disorders, Harm Reduction, Stigma, Human Rights, Health Equity, Health Professions Education

## Abstract

**Background:**

Sex workers, particularly those who use drugs, experience intersecting stigma and structural barriers that can worsen mental health and sexual health outcomes and limit access to affirming care. Although lived and living expertise is increasingly recognized as valuable in clinician education, perspectives from sex workers who use drugs remain underrepresented in mental health clinician training.

**Methods:**

In April 2025, a virtual, 1-h educational discussion was delivered to interdisciplinary Veterans Affairs mental health staff and trainees. The session was led by a bisexual, cisgender male sex worker who was a United States Army Veteran in recovery and moderated by a social worker with lived expertise. Topics included defining sex work, the role of substances in sex work, harm reduction strategies, and person-centered care. Anonymous pre-/post-surveys included items adapted from a previously published survey instrument and used 5-point Likert scale responses to assess attendee characteristics and changes in knowledge and attitudes related to sex workers.

**Results:**

Thirty-six clinicians attended; 24 completed both surveys. Most were physicians (37.5%) or psychologists (20.8%). Half (50.0%) had previously cared for a sex worker, yet only 29.2% reported prior formal education on sex work. Post-session surveys showed increased self-rated knowledge about sex work (2.2 ± 1.1 to 3.2 ± 0.8), improved recognition of sex workers’ rights to access mental health care (4.7 ± 1.0 to 5.0 ± 0.2), and greater awareness of healthcare barriers encountered by sex workers (4.0 ± 1.0 to 4.3 ± 0.8). Willingness to provide care was high at baseline and remained high post-session (5.0 ± 0.2 to 5.0 ± 0.2).

**Conclusions:**

A brief, Veteran-led discussion incorporating lived and living expertise was associated with meaningful short-term increases in clinician knowledge about sex work. Findings support integrating sex work-informed, trauma-informed content into routine clinician education and partnering with lived and living experience educators to improve relevance and reduce stigma.

**Supplementary Information:**

The online version contains supplementary material available at 10.1186/s12909-026-09113-x.

## Introduction

Sex workers, particularly those who use drugs, experience intersecting stigma and structural barriers that can worsen mental health and sexual health outcomes and limit access to affirming care [15, 18, 25, 36]. In healthcare settings, these barriers may affect disclosure, trust, continuity, and willingness to seek services [8, 9, 24]. Despite these implications for clinical practice, formal education on sex work remains limited in mental health and substance use training programs, and lived and living expertise (LLE) from people engaged in sex work is rarely centered in clinician education [2, 14, 28].

Centering LLE may be especially important in this area because sex workers have frequently reported that academic, public health, and clinical narratives mischaracterize their experiences and priorities, particularly when training is developed without meaningful partnership from people directly affected [14, 23, 31]. LLE-informed education can improve the relevance, credibility, and accuracy of educational content,challenge stereotypes that are often reinforced in traditional training; and help clinicians translate concepts such as autonomy, dignity, stigma, and trauma-informed care into practice [4, 6, 17, 20, 28].

Sex work is heterogeneous and encompasses a wide range of activities and contexts, including street-based sex work, escorting, erotic dancing, pornography, online cam work, kink and fetish work, and telephone sex [33]. These contexts differ in visibility, autonomy, safety considerations, exposure to stigma, and associated mental and physical health risks [23, 29, 34]. Clinician education should therefore reflect the diversity of sex work rather than treat sex workers as a single, uniform population [14, 33].

Importantly, barriers to person-centered care are not only interpersonal. Criminalization, documentation practices, clinic workflows, eligibility requirements, and other institutional and policy factors can amplify stigma, shape public and professional perceptions of sex work, deter disclosure, and reduce access to respectful care [9, 18, 24, 25, 36]. Clinician education is therefore one modifiable organizational lever through which health systems can reduce discriminatory treatment, improve sexual health-related care experiences, and strengthen accountability for affirming care [4, 26, 28].

Experiences in sex work are also heterogeneous with respect to choice, coercion, and structural vulnerability [11, 28, 32]. Some people engage in sex work voluntarily for reasons that may include income, autonomy, flexibility, safety afforded by online platforms, or sexual expression, whereas others experience coercion, exploitation, or trafficking [11, 13, 28, 29, 32]. Distinguishing consensual sex work from trafficking is essential for accurate, non-stigmatizing clinical understanding [28, 32]. Public health and clinical narratives have often framed sex work primarily through a stigmatizing lens, focusing on the nature of the work rather than the conditions under which it occurs and the person behind the work [22, 27, 29, 35]. These narratives can reinforce inaccurate stereotypes, such as assumptions that all sex workers are coerced, controlled by a third party, use methamphetamine or injection drugs, are unhoused, cannot have families, or engage in unsafe sex [1, 11, 28, 33].

The relationship between sex work and substance use is also complex. The assumption that all sex workers use or have used illicit substances is inaccurate [1, 3]. Substance use in sex work contexts is heterogeneous and may involve alcohol, methamphetamine, opioids, or other substances used for reasons including coping with trauma and stress, managing work demands, enhancing dissociation, or social and occupational contexts [3, 5, 30]. When substance use co-occurs with sex work, individuals may experience additional stigma, trauma exposure, violence, infection and overdose risk, and barriers to care [3, 7, 9, 30]. Because many sexual health services, including HIV and sexually transmitted infection (STI) screening, prevention counseling, and linkage to treatment, are delivered within or alongside mental health and substance use settings, clinician preparedness in these settings has direct implications for sexual health equity [9, 24, 28].

Few published studies have examined sex-worker-led education tailored to mental health clinicians, particularly within the Veterans Health Administration (VHA). This quality improvement (QI) initiative evaluated whether a brief, Veteran-led discussion centered on LLE could improve clinician knowledge and attitudes relevant to providing affirming, nonjudgmental care for people engaged in sex work, including those who use drugs.

## Methods

### Design and setting

This project was conducted as a QI educational initiative to assess and strengthen clinician knowledge and attitudes related to caring for patients engaged in sex work and substance use. The initiative was implemented at the San Francisco Veterans Affairs (VA) Health Care System, which serves over 310,000 United States (US) Veterans and includes the San Francisco VA Medical Center and nine community-based outpatient clinics in downtown San Francisco, San Bruno, Oakland, Santa Rosa, Clearlake, Ukiah, and Eureka, California. This activity was reviewed using the US Department of VA Electronic Determination Aid (VAEDA) and was determined to constitute QI/program evaluation and not human subjects research (ID #57,682); therefore, the activity was not submitted to a local ethics committee/IRB for review and approval. Under US federal regulations governing human subjects research (38 CFR Part 16, the VA’s Common Rule), IRB review and informed consent requirements apply to research involving human subjects; this activity was classified as QI/program evaluation and not human subjects research. Participation in anonymous pre/post-education surveys was voluntary. No direct identifiers were collected.

### Context and participants

Eligible participants were interdisciplinary Addiction Recovery Treatment Services staff and trainees who attended a recurring substance use disorder seminar. Seminar attendance occurred through routine educational programming and standardized seminar announcements. No targeted recruitment beyond the usual seminar distribution process was conducted. Completion of the evaluation surveys was voluntary, and no incentives were offered.

### Educational session

A virtual, 1-h Veteran-led educational discussion was delivered in April 2025 during the recurring substance use disorder seminar, which had approximately 190 invitees. The session was moderated by a licensed clinical social worker with lived expertise of sex work and substance use. Content was co-developed by the moderator and Veteran speaker and addressed: (1) defining sex work, (2) the role of substances in sex work, (3) harm reduction strategies, and (4) person-centered care approaches. Attendees could submit written questions via the virtual meeting chat, which were addressed throughout the session and during a question-and-answer period at the end. Additional educational resources, such as webinar opportunities, handouts, and videos, were shared after the session.

### Program evaluation data collection

Seminar leadership approved administration of brief, anonymous pre- and post-session surveys as part of routine educational evaluation. Attendees accessed surveys via a quick response code linked to Qualtrics.

### Measures

Pre- and post-session surveys were developed in Qualtrics (Additional Files 1–2). Survey items assessing knowledge, attitudes, human rights-related beliefs, and willingness to provide care were adapted from Ma and Loke and were not independently validated for this specific population or setting [19]. Likert-type items used 5-point response scales, with anchors varying by item type. For familiarity, knowledge, and interest items, higher scores indicated greater familiarity, knowledge, or interest (e.g., 1 = not at all familiar/no knowledge at all/not at all interested; 5 = very familiar/very knowledgeable/very interested). For agreement-based items, responses ranged from 1 = strongly disagree to 5 = strongly agree. Accordingly, higher scores reflected greater endorsement of positively framed items (e.g., rights to care, willingness to provide care, ability to recognize barriers) and greater endorsement of negatively framed items for reverse-valence statements such as ‘sex work is immoral’ and ‘sex workers who acquire HIV or sexually transmitted infections deserve no sympathy.’ Brief sociodemographic questions were adapted from Hughes et al. [10].

### Evaluation measures

The primary evaluation measure was the proportion of respondents reporting prior formal education on sex work. Secondary evaluation measures included pre/post changes in self-reported knowledge about sex work and willingness to provide care to sex workers.

### Analysis

Survey responses were summarized using descriptive statistics. Consistent with the QI purpose of this evaluation and the small sample size, findings were interpreted descriptively rather than inferentially. This analytic approach was intended to characterize short-term pre-/post-session patterns and inform local educational improvement. Statistical calculations were performed in Microsoft Excel (version 2208).

## Results

Thirty-six individuals attended the educational session; 24 participants completed both the pre- and post-session evaluation surveys. As shown in Table 1, respondents represented multiple disciplines, most commonly physicians (*n* = 9, 37.5%) and psychologists (*n* = 5, 20.8%). Most respondents identified as women (*n* = 15, 62.5%), and nearly half identified as White or European American (*n* = 11, 45.8%).

**Table 1:** Sociodemographic Characteristics of Survey Responders (*n* = 24)

Sociodemographics		N (%)
Discipline	Physician	9 (37.5)
	Psychologist	5 (20.8)
	Social worker	3 (12.5)
	Other	3 (12.5)
	Nurse	2 (8.3)
	Pharmacist	1 (4.2)
Age, years, mean ± SD		36.1 ± 8.1
Gender	Woman	15 (62.5)
	Man	5 (20.8)
	Non-binary or gender non-conforming	2 (8.3)
Transgender		2 (8.3)
Race/Ethnicity	White or European American	11 (45.8)
	Asian or Asian American	6 (25.0)
	Hispanic, Latinx, Latine, Latino, or Latina	4 (16.7)
	Black or African American	1 (4.2)
	Native Hawaiian or Other Pacific Islander	1 (4.2)

Baseline experience and training related to sex work varied (Table 2). Half of respondents (*n* = 12, 50.0%) reported having previously provided care to a sex worker; however, fewer than one-third (*n* = 7, 29.2%) reported any prior formal education on sex work. Baseline familiarity with California sex work laws was low (mean 1.7 ± 1.0). Across the pre/post-session evaluation surveys, the largest descriptive changes were observed in self-rated knowledge about sex work (mean 2.2 ± 1.1 pre-session to 3.2 ± 0.8 post-session), recognition of sex workers’ right to access high-quality mental health care (mean 4.7 ± 1.0 to 5.0 ± 0.2), and perceived ability to recognize barriers to healthcare encountered by sex workers (mean 4.0 ± 1.0 to 4.3 ± 0.8). Several rights-based and non-stigmatizing attitudes were favorable at baseline and remained favorable post-session. Respondents strongly disagreed that sex workers who acquire HIV or STIs deserve no sympathy (mean 1.0 ± 0.2 to 1.0 ± 0.0) and that sex work is immoral (mean 1.2 ± 0.5 to 1.2 ± 0.7). Willingness to provide care was also high both before and after the session (mean 5.0 ± 0.2 to 5.0 ± 0.2).

**Table 2:** Pre- and Post-Educational Session Survey Results (*n* = 24)

Theme	Question	Pre-Survey		Post-Survey
Knowledge of sex workers and laws	Have you ever met a sex worker? N (%)	Yes	15 (62.5)	
		No	3 (12.5)	
	Do you personally know someone who engages in sex work? N (%)	Yes	7 (29.2)	
		No	15 (62.5)	
	Have you ever provided care to a sex worker? N (%)	Yes	12 (50.0)	
		No	6 (25.0)	
	Have you ever attended a lecture, educational course, or community forum about sex work? N (%)	Yes	7 (29.2)	
		No	17 (70.8)	
	How familiar are you with sex work laws in California? (1 = not at all familiar; 5 = very familiar) mean ± SD (range)	1.7 ± 1.0 (1–4)		
	How would you rate your level of knowledge about sex work? (1 = no knowledge at all; 5 = very knowledgeable) mean ± SD (range)	2.2 ± 1.1 (1–4)		3.2 ± 0.8 (1–5)
	How interested are you in learning more about sex work? (1 = not at all interested; 5 = very interested) mean ± SD (range)	4.5 ± 1.1 (1–5)		4.5 ± 0.8 (3–5)
Attitudes towards sex workers, HIV, and STIs	Sex work should be legalized. (1 = strongly disagree; 5 = strongly agree) mean ± SD	4.0 ± 0.9 (2–5)		4.2 ± 0.9 (2–5)
	Sex work is immoral. (1 = strongly disagree; 5 = strongly agree) mean ± SD (range)	1.2 ± 0.5 (1–3)		1.2 ± 0.7 (1–4)
	Sex workers who acquire HIV or STIs deserve no sympathy. (1 = strongly disagree; 5 = strongly agree) mean ± SD (range)	1.0 ± 0.2 (1–2)		1.0 ± 0.0 (1–1)
	Sex workers should be given free condoms to reduce the spread of HIV/STIs. (1 = strongly disagree; 5 = strongly agree) mean ± SD (range)	4.8 ± 0.9 (1–5)		4.8 ± 0.9 (1–5)
Support for sex workers’ human rights	Sex workers have the right to non-discrimination and equal treatment. (1 = strongly disagree; 5 = strongly agree) mean ± SD (range)	4.7 ± 0.9 (1–5)		4.8 ± 0.9 (1–4)
	Sex workers have the right to information and education that may affect their well-being. (1 = strongly disagree; 5 = strongly agree) mean ± SD (range)	4.8 ± 0.9 (1–5)		5.0 ± 0.2 (4–5)
	Sex workers have the right to access the highest attainable standard of physical health care. (1 = strongly disagree; 5 = strongly agree) mean ± SD (range)	4.7 ± 1.0 (1–5)		4.7 ± 0.9 (1–5)
	Sex workers have the right to access the highest attainable standard of mental health care. (1 = strongly disagree; 5 = strongly agree) mean ± SD (range)	4.7 ± 1.0 (1–5)		5.0 ± 0.2 (4–5)
	Sex workers have the right to access basic necessities (e.g., housing, food, clothing) needed for an adequate standard of living. (1 = strongly disagree; 5 = strongly agree) mean ± SD (range)	4.8 ± 0.9 (1–5)		4.8 ± 0.7 (2–5)
Willingness to treat sex workers	I am willing to provide care to sex workers. (1 = strongly disagree; 5 = strongly agree) mean ± SD (range)	5.0 ± 0.2 (4–5)		5.0 ± 0.2 (4–5)
	I can recognize potential barriers to healthcare that might be encountered by sex workers. (1 = strongly disagree; 5 = strongly agree) mean ± SD (range)	4.0 ± 1.0 (2–5)		4.3 ± 0.8 (2–5)

Percentages may not total 100% because survey items were optional, and some respondents skipped individual questions

*HIV* human immunodeficiency virus, *STI* sexually transmitted infection

## Discussion

This QI educational initiative suggests that a brief, virtual, Veteran-led discussion incorporating LLE may strengthen clinicians’ understanding of sex work and support meaningful short-term changes in self-reported attitudes among interdisciplinary mental health clinicians. The largest descriptive changes were observed in self-rated knowledge about sex work, recognition of sex workers’ right to access high-quality mental health care, and perceived ability to identify barriers encountered in healthcare settings. These domains are directly relevant to sexual health equity because mental health and substance use services often function as entry points to HIV/STI prevention, screening, and linkage to care.

These findings are consistent with prior educational literature suggesting that brief stigma-reduction and lived-experience-informed interventions can improve self-reported knowledge and attitudes in the short term, even when long-term behavior change is not yet established [6, 16, 21, 28]. At the same time, the present findings should be interpreted as early educational signals rather than evidence of sustained practice change. Several items were already favorable at baseline, suggesting ceiling effects for some attitudes and willingness-to-care measures. In this context, the observed changes are still meaningful because they occurred after a single brief session and in a sample that had substantial clinical exposure to sex workers but limited prior formal education on the topic.

Sex workers experience elevated mental health and substance use needs and are exposed to occupational and structural risks, including violence, criminalization, and discrimination [12, 22]. These barriers can deter disclosure, limit engagement, and worsen inequities in access to sexual and mental health care [22, 28]. Clinician attitudes and biases may influence whether sexual health needs are assessed, whether HIV/STI testing is offered, and whether harm reduction and supportive resources are discussed in a respectful, nonjudgmental manner [4, 23, 28]. Taken together, these realities help explain why educational interventions on sex work are not peripheral, but directly relevant to equitable care delivery in mental health and substance use settings.

A notable finding in this initiative was the mismatch between clinicians’ prior exposure to sex workers in practice and their limited prior formal education on sex work. Half of respondents reported having previously provided care for a sex worker, yet fewer than one-third had ever received any formal education on the topic. This gap likely leaves clinicians to rely on assumptions, informal learning, or broader cultural narratives when navigating sexual health counseling, trauma-informed support, person-centered goal setting, and stigma-sensitive communication. The findings therefore support routine onboarding and continuing education that more explicitly addresses sex work, sexual health, substance use, stigma, and structural determinants of care [14, 28].

A central strength of this intervention was the prominent role of LLE. LLE-informed education may contribute something meaningfully different from conventional didactic approaches by providing contextual nuance, challenging stereotypes reinforced through outsider narratives, and increasing the credibility and practical relevance of the content for clinicians. In the present initiative, the participation of a Veteran speaker may have further strengthened salience within a VHA context through shared cultural understanding and peer credibility. By centering LLE, the session may have helped clinicians translate abstract concepts such as stigma, autonomy, dignity, and structural barriers into more concrete and person-centered approaches to care.

Embedding these educational approaches into health systems may therefore offer a pragmatic strategy to reduce stigma and improve care experiences for people engaged in sex work, including those who also experience substance use and related health inequities. Educational efforts should not stand alone, however. Their impact is likely to be strengthened when paired with system-level supports such as documentation guidance, referral pathways, protected educational time, and structures for equitable partnership with lived-experience educators.

Future iterations should evaluate whether repeated or longitudinal training leads to sustained changes in clinician behaviors and care delivery, including sexual health screening practices, referral patterns, documentation language, and linkage to HIV/STI prevention and treatment. Implementation across diverse clinical settings, including rural and lower-resourced sites, would also help clarify generalizability and identify context-specific barriers and facilitators. Overall, the findings support continued development of sex work-informed, trauma-informed clinician education that is co-developed and co-led with people with LLE.

### Educational implications

This Veteran-led, LLE-informed educational intervention has several implications for health professions education in mental health, substance use treatment, and sexual health settings. First, findings support integrating sex work-informed content into clinician onboarding and continuing education. Participants entered the session with limited prior formal education despite clinical exposure to sex workers, underscoring a gap that is unlikely to close without protected educational time and clear expectations within training programs. Educational content should be trauma-informed, culturally responsive, and harm reduction-oriented, with an explicit focus on reducing stigma and strengthening clinician skills for nonjudgmental care, appropriate sexual health screening, prevention counseling, and referrals.

Second, results reinforce the importance of meaningful partnership with people with LLE in developing and delivering education. Training programs should operationalize this partnership through standardized processes for recruitment, equitable compensation, and role protections to improve curriculum relevance, credibility, and reduce tokenism. Programs should also provide trauma-informed facilitation supports for LLE educators, including clear confidentiality expectations, boundaries, options to decline questions that feel unsafe or stigmatizing, and attention to the possibility of re-traumatization when discussing criminalized or stigmatized work.

Third, education should include practical implementation supports that help clinicians translate training concepts into consistent clinical behaviors. Examples include structured referral resources for HIV/STI prevention, testing, and treatment; harm reduction resources; and confidentiality- and documentation-focused teaching points that minimize unintended disclosure or stigmatizing language in the medical record. Where feasible, programs can reinforce learning with brief scripts, referral lists, point-of-care resources, and documentation guidance for routine clinical use.

Finally, education programs should evaluate whether training leads to sustained improvements in learner outcomes and clinical practice. Evaluations can incorporate measures of stigma, learner confidence and skills, and patient-reported experiences of respect and autonomy, as well as uptake of sexual health services (e.g., HIV/STI testing and linkage) where feasible. Future educational efforts should also prioritize inclusion of diverse lived-experience perspectives (e.g., women, transgender and gender-diverse individuals, Black, Indigenous, and People of Color [BIPOC], and immigrant and migrant sex workers) to reflect the heterogeneity of sex work and strengthen curriculum relevance across settings.

### Strengths and limitations

Strengths of this initiative include its real-world implementation within routine clinical education, its interdisciplinary audience, and its Veteran-led, co-developed format centered on LLE. The session addressed a topic that many clinicians are likely to encounter in practice but for which few respondents reported prior formal education, increasing the practical relevance of the findings.

This was a single-site educational QI initiative with a small sample, no comparison group, and a short follow-up window; we did not assess long-term knowledge retention or downstream changes in clinical practice. A single, one-hour session may be insufficient to meaningfully shift deeply held biases without ongoing reinforcement. Participation was voluntary and may reflect self-selection of clinicians already motivated to learn about sex work, which may limit generalizability. The session was delivered in an urban setting with proximity to academic institutions and included a diverse mix of trainees and staff; results may differ in rural or lower-resourced settings or among clinicians with different baseline exposure to sex work.

Evaluation measures relied on self-reported survey responses, which are subject to social desirability bias, particularly for stigmatized topics. Survey items were adapted from prior instruments rather than validated specifically for this population and setting. Twelve of 36 attendees did not complete both surveys, and all items were optional; these factors may have introduced nonresponse bias and reduced precision. In addition, several items showed favorable baseline responses, limiting sensitivity to detect change. Finally, while this session centered a Veteran with LLE, inclusion of additional Veteran educators with diverse identities and experiences, including women, transgender and gender-diverse individuals, BIPOC, and immigrant or migrant sex workers, would strengthen representativeness and broaden the perspectives reflected in future training.

## Conclusion

This QI educational session suggests that a brief, Veteran-led discussion incorporating LLE of sex work and substance use can produce meaningful short-term improvements in clinician knowledge and selected attitudes within a VHA mental health training context. Participants demonstrated increased understanding of sex work and greater recognition of sex workers’ rights to access mental health services, despite limited prior formal education on the topic. These findings support integrating sex work- and trauma-informed content into routine workforce development and doing so in partnership with people with LLE as a core educational strategy rather than an optional enhancement. Near-term program evaluation should assess whether repeated training improves knowledge retention, clinician confidence, and translation into practice. Longer-term work should examine whether these educational approaches influence documentation practices, sexual health screening and referral behaviors, and linkage to harm reduction and sexual health services across diverse settings.

## Supplementary Information

Additional file 1.

Additional file 2.

## Data Availability

The dataset supporting the conclusions of this article is included within the article.
