# Supplementary material for: Incorporating a Veteran’s lived and living expertise of sex work and substance use into clinician education
Source: BMC Med Educ. 2026 Apr 6;26:807. doi: 10.1186/s12909-026-09113-x (PMC13217652; doi:10.1186/s12909-026-09113-x)
Supplement: Supplementary file 1 — Additional file 1. [file 12909_2026_9113_MOESM1_ESM.pdf]

UCSF
University of California
San Francisco

Pre-Session_Knowledge, Attitudes, and Willingness to Care for Sex Workers

Survey Flow

Standard: Sex_work_pre (26 Questions)

Page Break

UCSF University of California San Francisco

Start of Block: Sex_work_pre

Sex_work_intro Thank you for your interest in joining this 1-hour, Veteran-led discussion on sex work and substance use.   As described by the National Harm Reduction Coalition, sex work is the provision of sexual services or performances by one person (sex worker) for another person (client or observer) in exchange for money or other markers of economic value (e.g., food, shelter, drugs). Sex work is an umbrella term that includes escort services, trade sex, porn performance, dancing, phone, webcam/internet, survival-based, street-based, BDSM and other kink-focused work, magazine, film/video, and more.   Please complete this brief survey before attending the session. All responses are optional and anonymous.

Sex_work_met Have you ever met a sex worker?

- ◯ Yes (1)
- ◯ No (2)
- ◯ Not sure (3)

Sex_work_know Do you personally know someone who engages in sex work?

- ◯ Yes (1)
- ◯ No (2)
- ◯ Not sure (3)

Sex_work_care Have you ever provided care to a sex worker?

- ◯ Yes (1)
- ◯ No (2)
- ◯ Not sure (3)

Sex_work_edu Have you ever attended a lecture, educational course, or community forum about sex work?

- ◯ Yes (1)
- ◯ No (2)
- ◯ Not sure (3)

Sex_work_laws How familiar are you with sex work laws in California? Drag the slider bar to record your response.

	1 = Not at all familiar			5 = Very familiar
	1	2	3	4	5
()					

Page Break

Sex_work_knowledge How would you rate your level of knowledge about sex work? Drag the slider bar to record your response.

1 = No knowledge at all 5 = Very knowledgeable

	1	2	3	4	5
()					

Sex_work_edu_interes How interested are you in learning more about sex work? Drag the slider bar to record your response.

1 = Not at all interested 5 = Very interested

	1	2	3	4	5
()					

Page Break

Section_heading **For this section, please select your level of agreement with the following statements. Drag the slider bar to record your response.**

Sex_work_legal Sex work should be legalized.

1 = Strongly disagree 5 = Strongly agree

	1	2	3	4	5
()					

Sex_work_immoral Sex work is immoral.

1 = Strongly disagree 5 = Strongly agree

	1	2	3	4	5
()					

Sex_work_STI Sex workers who acquire HIV or sexually transmitted infections (STIs) deserve no sympathy.

1 = Strongly disagree 5 = Strongly agree

	1	2	3	4	5
()					

Sex_work_condoms Sex workers should be given free condoms to reduce the spread of HIV/STIs.

	1 = Strongly disagree	5 = Strongly agree
	1 2 3 4 5	
()		

Sex_work_equal Sex workers have the right to non-discrimination and equal treatment.

	1 = Strongly disagree	5 = Strongly agree
	1 2 3 4 5	
()		

Sex_work_info Sex workers have the right to information and education that may affect their well-being.

	1 = Strongly disagree	5 = Strongly agree
	1 2 3 4 5	
()		

Sex_work_physical Sex workers have the right to access the highest attainable standard of physical health care.

	1 = Strongly disagree	5 = Strongly agree
	1 2 3 4 5	

()	

Sex_work_mental Sex workers have the right to access the highest attainable standard of mental health care.

1 = Strongly disagree 5 = Strongly agree

	1	2	4	5
()				

Sex_work_basic Sex workers have the right to access basic necessities (e.g., housing, food, clothing) needed for an adequate standard of living.

1 = Strongly disagree 5 = Strongly agree

	1	2	3	3	4	5
()						

Sex_work_care I am willing to provide care to sex workers.

1 = Strongly disagree 5 = Strongly agree

	1	2	3	4	5
()					

Sex_work_barriers I can recognize potential barriers to healthcare that might be encountered by sex workers.

	1 = Strongly disagree	5 = Strongly agree
	1 2 3 4 5	
()		

Page Break

Demo_discipline What is your **discipline**?

- Nurse (6)
- Peer specialist (5)
- Pharmacist (3)
- Physician (4)
- Psychologist (2)
- Social worker (1)
- Other (7)

---

*

Demo_age What is your **age** in years?

____

Demo_gender In research and quality improvement work, we often must present demographic information in categories. We understand these labels are limiting. If you had to select one of the options below, which one best describes your **gender identity**?

- ◯ Man (1)
- ◯ Woman (4)
- ◯ Agender (7)
- ◯ Gender fluid (8)
- ◯ Gender queer (9)
- ◯ Gender questioning (10)
- ◯ Māhū, or muxe, or two spirit (11)
- ◯ Non-binary/ Gender non-conforming (2)
- ◯ Another term best describes my gender: (5) ____
- ◯ Prefer not to answer (6)

Demo_trans Do you identify as **transgender**?

- ◯ Yes (1)
- ◯ No (2)
- ◯ Prefer not to answer (3)

University of California
San Francisco

Demo_race What label(s) best describe your **race/ethnicity**?

- ☐ American Indian, Native American, or Alaska Native (1)
- ☐ Arab, Middle Eastern, or North African (5)
- ☐ Asian or Asian American (2)
- ☐ Black or African American (3)
- ☐ Hispanic, Latinx, Latine, Latino, or Latina (4)
- ☐ Native Hawaiian or Other Pacific Islander (6)
- ☐ White or European American (7)
- ☐ Some other race, ethnicity, or origin: (10) ____
- ☐ Don't know (8)
- ☐ Prefer not to answer (9)

Demo.sexorien What term best describes your **sexual orientation**?

- ○ Asexual or aromantic (7)
- ○ Bisexual (8)
- ○ Demisexual (9)
- ○ Gay (10)
- ○ Lesbian (2)
- ○ Pansexual (3)
- ○ Queer (4)
- ○ Questioning (11)
- ○ Sexually fluid (12)
- ○ Straight or Heterosexual (1)
- ○ Another term best describes my sexual orientation: (5) ____
- ○ Prefer not to answer (6)

End of Block: Sex_work_pre
