# Supplementary material for: Incorporating a Veteran’s lived and living expertise of sex work and substance use into clinician education
Source: BMC Med Educ. 2026 Apr 6;26:807. doi: 10.1186/s12909-026-09113-x (PMC13217652; doi:10.1186/s12909-026-09113-x)
Supplement: Supplementary file 2 — Additional file 2. [file 12909_2026_9113_MOESM2_ESM.pdf]

Post-Session_Knowledge, Attitudes, and Willingness to Care for Sex Workers

Survey Flow

Standard: Sex_work_post (15 Questions)

Page Break

Start of Block: Sex_work_post

Sex_work_intro Thank you for joining this 1-hour, Veteran-led discussion on sex work and substance use.   As described by the National Harm Reduction Coalition, sex work is the provision of sexual services or performances by one person (sex worker) for another person (client or observer) in exchange for money or other markers of economic value (e.g., food, shelter, drugs). Sex work is an umbrella term that includes escort services, trade sex, porn performance, dancing, phone, webcam/internet, survival-based, street-based, BDSM and other kink-focused work, magazine, film/video, and more.   Please complete this brief survey after attending the session. All responses are optional and anonymous.

Sex_work_knowledge How would you rate your level of knowledge about sex work? Drag the slider bar to record your response.

	1 = No knowledge at all				5 = Very knowledgeable
	1	2	3	4	5
()					

Sex_work_edu_interes How interested are you in learning more about sex work? Drag the slider bar to record your response.

	1 = Not at all interested				5 = Very interested
	1	2	3	4	5
()					

Page Break

Section_heading **For this section, please select your level of agreement with the following statements. Drag the slider bar to record your response.**

Sex_work_legal Sex work should be legalized.

1 = Strongly disagree 5 = Strongly agree

	1	2	3	4	5
()					

Sex_work_immoral Sex work is immoral.

1 = Strongly disagree 5 = Strongly agree

	1	2	3	4	5
()					

Sex_work_STI Sex workers who acquire HIV or sexually transmitted infections (STIs) deserve no sympathy.

1 = Strongly disagree 5 = Strongly agree

	1	2	3	4	5
()					

University of California
San Francisco

Sex_work_condoms Sex workers should be given free condoms to reduce the spread of HIV/STIs.

1 = Strongly disagree 5 = Strongly agree

	1	2	3	4	5
()					

Sex_work_equal Sex workers have the right to non-discrimination and equal treatment.

1 = Strongly disagree 5 = Strongly agree

	1	2	3	4	5
()					

Sex_work_info Sex workers have the right to information and education that may affect their well-being.

1 = Strongly disagree 5 = Strongly agree

	1	2	3	4	5
()					

Sex_work_physical Sex workers have the right to access the highest attainable standard of physical health care.

1 = Strongly disagree 5 = Strongly agree

1 2 3 4 5

()	

Sex_work_mental Sex workers have the right to access the highest attainable standard of mental health care.

1 = Strongly disagree 5 = Strongly agree

	1	2	4	5
()				

Sex_work_basic Sex workers have the right to access basic necessities (e.g., housing, food, clothing) needed for an adequate standard of living.

1 = Strongly disagree 5 = Strongly agree

	1	2	3	3	4	5
()						

Sex_work_care I am willing to provide care to sex workers.

1 = Strongly disagree 5 = Strongly agree

	1	2	3	4	5
()					

Sex_work_barriers I can recognize potential barriers to healthcare that might be encountered by sex workers.

1 = Strongly disagree 5 = Strongly agree

	1	2	3	4	5
()					

Page Break

End of Block: Sex_work_post
